# Ginkgolide C alleviates atherosclerosis by activating LAMP-2A to enhance chaperone-mediated autophagy and promote NLRP3 inflammasome degradation

**DOI:** 10.3389/fphar.2025.1658725

**Published:** 2025-11-07

**Authors:** Rui Zhang

**Affiliations:** Department of Pharmacy, Shandong Provincial Hospital Affiliated to Shandong First Medical University, Jinan, Shandong, China

**Keywords:** ginkgolide C, atherosclerosis, NLRP3 inflammasome, chaperone-mediated autophagy, LAMP-2A

## Abstract

**Introduction:**

The NLRP3 inflammasome/IL-1β-dependent inflammatory response serves as a critical factor and key trigger in exacerbating atherosclerosis (AS), whereas chaperone-mediated autophagy (CMA) recognizes and degrades the NLRP3 inflammasome. Targeting this pathway represents a more nuanced and targeted anti-inflammatory strategy to mitigate AS progression. As a key bioactive component derived from *Ginkgo biloba* leaves, Ginkgolide C (GC) possesses notable anti-inflammatory effects and confers protection against myocardial and cerebral ischemia-reperfusion injuries. The current research aimed to investigate whether GC could exert protective effects against AS and to elucidate its potential underlying mechanisms.

**Method:**

This study established both *in vivo* (high-fat diet/vitamin D3-induced atherosclerotic mouse model) and *in vitro* (LPS/ATP-stimulated RAW264.7 macrophage injury model) systems. *In vivo* evaluations included: H&E and Oil Red O staining for atherosclerotic lesion assessment; biochemical detection for lipid profiles; transmission electron microscopy for autophagic structure observation; immunohistochemistry and immunofluorescence for CMA regulator (LAMP-2A), NLRP3 inflammasome as well as key pro-inflammatory cytokines such as IL-1β, IL-18, and TNF-α. *In vitro* analyses comprised: MTT assay for cell viability; ELISA for quantifying inflammatory cytokine secretion; Western blotting for LAMP-2A, NLRP3 inflammasome, and NF-κB, MAPK signaling pathways molecules. LAMP-2A knockdown was conducted using siRNA to validate the CMA-dependent mechanisms underlying GC’s effects.

**Result:**

Our results demonstrate that GC potentiated CMA activity in macrophages, leading to promoted degradation of the NLRP3 inflammasome via the lysosomal pathway. This process effectively suppressed the NLRP3 inflammasome/IL-1β-driven inflammatory cascade, ultimately attenuating atherosclerotic progression.

**Conclusion:**

GC alleviates AS via a novel LAMP-2A-dependent mechanism that enhances protein clearance and suppresses NLRP3 inflammation, providing a targeted alternative to broad immunosuppression. These results establish GC as a promising therapeutic candidate and prompt further studies on its clinical efficacy and applicability in other chronic inflammatory diseases.

## Introduction

Atherosclerosis (AS) is widely recognized as the pathological cornerstone of cardiovascular disorders, imposing a significant global health burden due to its elevated morbidity and mortality rates (Pedro-Botet et al., 2020; Doran, 2022). It is now characterized as a chronic vascular inflammatory disorder stemming from the intricate interplay of metabolic and immunological factors. Its pathogenesis is centrally linked to the progressive accumulation and oxidative modification of lipoprotein particles within the subendothelial space, which triggers a cascade of inflammatory responses and plaque formation in arterial walls (Bugger and Zirlik, 2021). While current evidence indicates that plaque progression represents a potentially modifiable phase in AS development, the intricate molecular pathways governing lesion evolution remain incompletely characterized (Libby, 2021).

Accumulating evidence indicates that macrophage autophagy promotes cholesterol efflux, suppresses inflammatory mediators activation, and enhances lysosome-dependent degradation pathways, establishing it as a promising target for attenuating AS progression (Theofilis et al., 2023; Mackay et al., 2024). As key immune effectors originating from monocytic precursors, macrophages ubiquitously infiltrate all developmental phases of atherosclerotic lesions and actively participate in inflammatory pathogenesis via sustained secretion of pro-inflammatory cytokines and chemokines. Macrophage foam cell formation exacerbates plaque lipid accumulation, inflammatory activation, and lesion instability in AS (Chen et al., 2022). Consequently, therapeutic strategies targeting the reduction of intracellular lipid deposition in macrophage-derived foam cells and attenuation of pro-inflammatory signaling pathways have emerged as a critical focus for AS management. Recent studies have identified the autophagy-lysosome system as a key regulatory mechanism modulating atherosclerotic pathogenesis, offering promise for novel therapeutic strategies (Mameli et al., 2022).

Lysosomes, serving as the central degradative organelles of eukaryotic cells, coordinate the processing and recycling of damaged or dysfunctional cellular components through specialized enzymatic machinery (Deretic, 2021). Autophagic pathways are classified into three mechanistically distinct subtypes based on their substrate delivery mechanisms to lysosomes: macroautophagy, microautophagy and chaperone-mediated autophagy (CMA), which mediates the selective translocation of substrate proteins containing a KFERQ-like motif. CMA has been recognized as a critical quality control mechanism for maintaining proteostasis in mammalian cells. As a highly selective autophagic process, CMA specifically targets cytosolic proteins harboring a conserved KFERQ-like pentapeptide motif. Unlike other autophagic pathways, CMA does not involve vesicle formation but instead relies on the Heat Shock Cognate 70 (Hsc70) chaperone complex for substrate recognition, unfolding, and translocation. This process depends critically on the lysosomal membrane receptor LAMP-2A, which directly binds the Hsc70-substrate complex (Xu and Wan, 2023). Moreover, LAMP-2A multimerization at the lysosomal membrane constitutes the active translocation complex, making it the rate-limiting step in CMA flux (Zhang et al., 2024). Therefore, genetic modulation of LAMP-2A expression is the most established method for precise CMA manipulation, highlighting its therapeutic potential in proteostasis-related diseases.

Recent research has reconceptualized AS as a chronic inflammatory disorder in which the NLRP3 inflammasome/interleukin-1β (IL-1β) cascade acts as a central driver of pathogenesis (Chen et al., 2021; Sharma and Kanneganti, 2021). As the upstream molecular platform responsible for IL-1β maturation, the NLRP3 inflammasome serves as a core regulator of innate immunity. Pathological activation of the NLRP3 inflammasome is evident from the earliest stages of AS, with persistent inflammasome hyperactivity and IL-1β hypersecretion occurring throughout disease progression. Tumor necrosis factor-alpha (TNF-α) serves as a priming signal that induces the transcriptional and post-translational mechanisms required for NLRP3 activation (Tall and Bornfeldt, 2023). Notably, strategies that promote the degradation of the assembled NLRP3 inflammasome-rather than directly neutralizing IL-1β or inhibiting upstream assembly-offer a more targeted and efficient anti-inflammatory approach (Xu and Núñez, 2023). In this pathway, TNF-α primes NLRP inflammasome formation, leading to Caspase-1 activation and subsequent cleavage and secretion of mature IL-1β, establishing a core inflammatory axis. In comparison to the proteasome or macroautophagy systems, CMA represents a highly specific protein quality control mechanism capable of selective substrate degradation. Growing evidence suggests that CMA modulates the NLRP3 inflammasome activation cascade and limits IL-1β production through targeted degradation of key inflammasome components. Accordingly, CMA dysfunction, particularly deficiency in the lysosomal receptor LAMP-2A, may accelerate atherosclerotic plaque progression through hyperactivation of the NLRP3/IL-1β axis and sustained vascular inflammation. Although the NLRP3 inflammasome is widely accepted as a contributor to AS, several conceptual and translational controversies remain. Ongoing debate concerns the dominant activation triggers and signaling hierarchy within vascular inflammation, and, more importantly, how to selectively inhibit NLRP3 without inducing systemic immunosuppression. These unresolved issues underscore the urgency of developing novel and precise mechanistic interventions.

The elucidated CMA regulatory mechanisms naturally extend to exploring clinically translatable modulators. Of particular interest are botanicals with historic neuroprotective applications, such as *Ginkgo biloba* extracts, whose terpenoid constituents exhibit structural compatibility with lysosomal membrane proteins critical for autophagy maturation (Noor et al., 2022). *Ginkgo biloba* has long been used in traditional Chinese medicine for its medicinal potential. Contemporary scientific investigations have substantiated that extracts derived from its leaves exhibit a range of pharmacological effects, including cardioprotection, neurovascular protection, and anticancer potential (Silva and Martins, 2022). Among its bioactive constituents, ginkgolide C (GC), a terpenoid compound isolated from the leaves of *G. biloba*, has garnered increasing interest. Our earlier studies have established GC as a compound with multifaceted bioactivities, demonstrating significant anti-inflammatory efficacy and protective effects against myocardial and cerebral ischemia-reperfusion injury (Zhang et al., 2018; Li et al., 2023). Based on these known protective properties of GC, we hypothesized that its atheroprotective effects are mediated through the activation of CMA-a selective degradation pathway critically involved in regulating inflammation and lipid metabolism, both central aspects of AS pathogenesis. AS represents the fundamental pathological basis of cardiovascular and cerebrovascular diseases related to ischemia-reperfusion injury, with strong evidence supporting a bidirectional pathophysiological crosstalk. However, the therapeutic potential of GC in modulating atherosclerotic progression through CMA-associated LAMP-2A induction remains unexplored.

Thus, the present study utilized integrated experimental approaches *in vivo* and *in vitro* to systematically evaluate the anti-atherosclerotic effects of GC, with a focus on elucidating its dual mechanism of action. Our results demonstrate that GC potentiates CMA activity in macrophages, leading to promoted degradation of the NLRP3 inflammasome, suppression of NLRP3/IL-1β-mediated inflammation, and ultimately conferring protection against atherosclerosis. This work provides a mechanistic basis for understanding AS pathogenesis and proposes CMA potentiation as a promising therapeutic strategy for the clinical management of AS and other chronic inflammatory diseases.

## Materials and methods

### Materials and reagents

GC (PubChem CID: 161120) and Atorvastatin (AVT, PubChem CID: 60823) were purchased from Sigma-Aldrich (Sigma, MO, United States). Anti-LAMP-2A, anti-IL-1β, anti-interleukin-18 (anti-IL-18), anti-TNF-α, anti-phosphorylated (p)-IκB-α, anti-NF-κB p65, anti-p-JNK, anti-p-ERK and IgG-HRP antibodies were purchased from Abcam (Cambridge, MA, United States). Anti-p-p38 MAPK and anti-NLRP3 were from Cell Signaling Technology (CST, Danvers, MA, United States). Anti-β-actin and anti-histone were purchased from Cloud-Clone Corp. (Wuhan, China). The following commercial reagents were used according to the manufacturers’ protocols: BCA protein quantitation assay system (P0010S), PVDF immunoblotting membranes (FFP28), SDS-PAGE gel casting kits (PG012) and enhanced chemiluminescence detection kits (ECL Plus, P0018M) were obtained from Beyotime Biotechnology (Shanghai, China).

### Animals experiments

4-6-week-old male C57BL/6 mice and age-matched male Apolipoprotein E-deficient (ApoE^−/−^) male mice were obtained from Huazhong Agricultural University (Wuhan, China) and Guangdong Yaokang Biotechnology Co., Ltd. (Guangzhou, China), respectively. All animals were housed under specific pathogen–free (SPF) conditions in a climate-controlled environment (20 °C–25 °C; 40%–60% humidity) under a standardized 12 h light/12 h dark cycle, with free access to food and water. All experimental procedures were conducted in strict accordance with protocols approved by the Ethics Committee of the Shandong Provincial Hospital Affiliated to Shandong First Medical University (approval no. 2022-661).

Mice were randomly assigned to 6 experimental groups (n = 6 per group): control group, AS model group, 2.5 mg/kg AVT intervention group and GC treatment groups (12, 24, and 48 mg/kg). Atherogenic induction was implemented in ApoE^−/−^ mice (AS model, AVT, and GC groups) through combinatorial pharmacological potentiation and dietary modulation: 1) Pre-induction delivery of cholecalciferol (300,000 IU/kg in 0.2 mL saline; Sigma-Aldrich V7000); 2) Sustained 16-week administration of HFHC diet (40% kcal from fat, TD.02011, Envigo) with defined macronutrient profile (17% protein, 43% carbohydrate, 0% added cholesterol). C57BL/6 mice fed a standard chow diet and provided purified water served as the control group and were maintained under the same environmental conditions (22 °C ± 1 °C, 55% ± 5% humidity, 12 h light/12 h dark cycle) for the entire 16-week period. AVT and GC were administered via tail vein injection for 7 consecutive days following model establishment. The dose of GC for *in vivo* use was selected based on preliminary pharmacokinetic and pharmacodynamic studies and with reference to doses established in our previous experimental models (Zhang et al., 2018; Li et al., 2023).

### Plaque assessment

Following fixation in 4% paraformaldehyde for 24 h, hearts containing the aortic root were cryoprotected in 30% sucrose/double-distilled water for 24 h, oriented vertically and embedded in OCT compound, then serially sectioned at a thickness of 10 μm through the aortic valve plane using a cryostat. Serial sections underwent histological evaluation using Oil Red O (O0625, Sigma-Aldrich, United States) and hematoxylin and eosin (H&E) staining to assess lipid deposition and general morphology, respectively. Mean plaque area was quantified using ImageJ software (version 1.8.0; National Institutes of Health, United States).

#### Transmission electron microscope (TEM) observation

Transverse sections of aortic roots were primarily fixed in 3% glutaraldehyde in 0.1 M phosphate buffer, pH 7.2, for 48–72 h, washed in phosphate-buffered saline (PBS), and subsequently embedded in Polybed 812 epoxy resin. Ultrathin sections (90 nm thick) were prepared using an ultramicrotome and examined using a JEM-2000EX transmission electron microscope.

### Immunohistochemistry

Serial aortic root sections were processed for immunohistochemical detection of IL-1β, IL-18, and TNF-α. After deparaffinization in xylene and rehydration through a graded ethanol series, antigen retrieval was performed using microwave irradiation in citrate buffer (pH 6.0) at 95 °C ± 0.5 °C for 15 min. Endogenous peroxidase activity was subsequently blocked with 3% H_2_O_2_/methanol, followed by nonspecific binding inhibition using 5% normal goat serum containing 0.3% Triton X-100. Primary antibodies (anti-IL-1β ab-259421, anti-IL-18 ab-240849, anti-TNF-α ab-9579; all 1:500, Abcam, United States) were applied at 4 °C overnight, followed by Solabio HRP-conjugated secondary antibody incubation and DAB chromogenic development, with interspersed PBS washes ensuring procedural specificity.

### Immunofluorescence staining

Immunofluorescence staining was conducted to evaluate the expression of LAMP-2A and NLRP3. Following fixation in ice-cold methanol for 10 min at −20 °C and three 5-min washes with PBS (pH 7.4), non-specific binding was blocked by incubation with blocking buffer containing 3% bovine serum albumin (BSA) and 0.1% Tween-20 for 1 h at room temperature. Staining for LAMP-2A: rabbit anti-LAMP-2A (ab-125068, Abcam, United States) and goat anti-rabbit IgG HRP (ab-205718, Abcam, United States). Staining for NLRP3: rabbit anti-NLRP3 (ab-263899, Abcam, United States) and goat anti-rabbit IgG HRP (ab-97051, Abcam, United States). Nuclei were counterstained with 4′,6-diamidino-2-phenylindole (DAPI; 0.1 μg/mL in PBS, 5 min; ab-285390, Abcam). Images were acquired using a Leica TCS SP8 system equipped with a ×40 oil immersion objective (NA 1.30) and LAS X software (version 3.7.4), employing standardized excitation/emission parameters (358/461 nm) with z-stack acquisition (1 μm step size).

### Measurement of blood lipid index in mice

After centrifuging murine whole blood at 2,000 × g for 20 min at 4 °C, the serum supernatant was collected and diluted twofold with phosphate-buffered saline (PBS, 1×). Levels of total cholesterol (TC), triglycerides (TG), low-density lipoprotein cholesterol (LDL-C), and high-density lipoprotein cholesterol (HDL-C) were quantified using a Beckman DXC700au clinical chemistry analyzer, calibrated traceable to NIST SRM 1951c, using endpoint enzymatic colorimetric assays with absorbance measured at 500 nm (37 °C ± 0.2 °C). All assays showed an intra-assay coefficient of variation <3%.

### Cells experiments

RAW264.7 murine macrophages were cultured in Dulbecco’s Modified Eagle Medium (DMEM, Gibco) supplemented with 10% heat-inactivated fetal bovine serum (FBS, Gibco) and dual antibiotics (100 U/mL penicillin +100 μg/mL streptomycin, Gibco) within a controlled tri-gas incubator (5% CO_2_/95% humidified air at 37 °C). To mimic pro-atherosclerotic inflammatory conditions, cells were stimulated with lipopolysaccharide (LPS; 100 ng/mL, 12 h) followed by adenosine triphosphate (ATP; 5 mM, 30 min).

Cells were assigned to 5 experimental groups (n = 3 per group): control group, baseline culture in standard DMEM without stimulation; model group, cells treated with LPS/ATP procedure; GC group, treatment with graded GC concentrations (1, 10, 100 mM in serum-reduced DMEM) for 24 h after LPS/ATP co-stimulation. The effective concentration of GC for *in vitro* experiments was empirically determined through initial cytotoxicity and dose-response assays, with the concentration ranges informed by our prior research (Zhang et al., 2018; Li et al., 2023).

### Reconstruction of LAMP-2A-silencing RAW264.7 cells

When RAW264.7 cells reached approximately 80%–85% confluence, cells in the control group were transfected with pGPU6/Hygro, while those in other groups were transfected with pGPU6/Hygro-LAMP-2A, using transfection reagent (GenePharma, Shanghai, China). Following transfection, cells were incubated for 24 h before subsequent GC pretreatment.

### Analysis of cell viability

Cell viability in RAW264.7 cultures was assessed using the MTT assay. After treatment, cells were incubated with MTT (5 mg/mL, final concentration) for 4 h at 37 °C. The medium was then carefully removed, and the resulting formazan crystals were dissolved in anhydrous dimethyl sulfoxide (DMSO) with orbital shaking at 300 rpm for 15 min. Absorbance was measured at 490 nm using a microplate reader and normalized to that of the control group.

### Detections of IL-1β, IL-18 and TNF-α

Cell culture supernatants were collected following GC treatment. IL-1β, IL-18, and TNF-α concentrations were determined by enzyme-linked immunosorbent assay (ELISA) kits (IL-1β 88-7013A-88, IL-18 KMC0181, TNF-α BMS607-3, ThermoFisher, United States).

### Western blot for LAMP-2A, NPRL3 inflammasome, NF-κB p65, p-IκB-α, p-JNK, p-ERK and p-p38 MAPK expressions in RAW264.7 cells

Total protein concentration was determined using a bicinchoninic acid (BCA) assay. Nuclear and cytoplasmic proteins were extracted using a commercial isolation kit (Nuclear-Cytoplasmic Protein Extraction Kit, P1250, Applygen Technologies, Beijing, China) according to the manufacturer’s instructions and previously described methods (Zhang et al., 2018). Equal amounts of protein (50 μg per lane) were separated by sodium dodecyl sulfate–polyacrylamide gel electrophoresis (SDS-PAGE) and transferred onto polyvinylidene fluoride (PVDF) membranes. After blocking with 5% non-fat milk, the membranes were incubated with primary antibodies (1:800 dilution) targeting LAMP-2A, NPRL3 inflammasome, NF-κB p65, p-IκB-α, p-JNK, p-ERK, and p-p38 MAPK at 37 °C for 4 h. The membranes were then incubated with horseradish peroxidase (HRP)-conjugated secondary antibodies (1:1000 dilution) at room temperature for 1 h. Protein bands were visualized using enhanced chemiluminescence (ECL) Plus reagent. Digital imaging and densitometric analysis were performed using a Gel Imaging System with Quantity One software (version 5.0, Bio-Rad Laboratories, Hercules, CA, United States).

### Statistical analysis

All datasets derived from triplicate experiments are presented as mean ± SD. Group comparisons were analyzed through one-way ANOVA with Bonferroni correction using GraphPad Prism 5.0 (GraphPad Software, Inc., San Diego, CA), with statistical significance defined at *P* < 0.05.

## Results

### GC inhibits atherosclerotic plaque progression and ameliorates blood lipid levels in AS model mice

**FIGURE 1:**
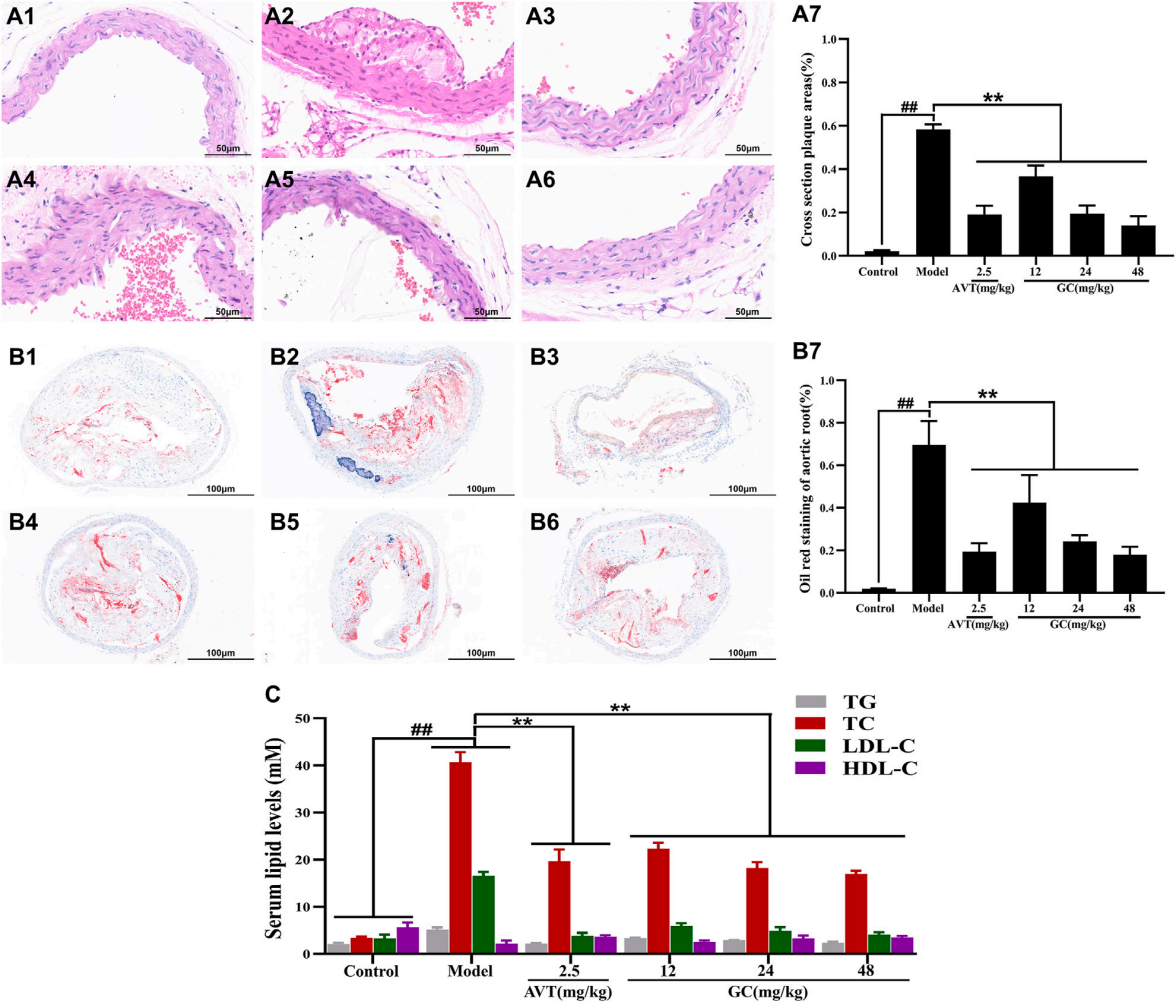
Effect of GC on atherosclerotic plaque formation and serum lipid levels in AS model mice. Light microscopy images (40 ×) of aortic roots stained with H&E **(A)** and oil red O **(B)**. (A1/B1) Control group, (A2/B2) model group, (A3/B3) 2.5 mg/kg AVT group, (A4/B4) 12 mg/kg GC group, (A5/B5) 24 mg/kg GC group, (A6/B6) 48 mg/kg GC group. (A7) Quantitative analysis of atherosclerotic plaque area based on H&E staining. (B7) Quantitative analysis of atherosclerotic plaque area based on oil red O staining. **(C)** Quantitative analysis of serum lipid levels. Data were expressed as mean ± SD (n = 6). ^##^ *P* < 0.01 vs. control group; ^∗^ *P* < 0.05, ^∗∗^ *P* < 0.01 vs. model group.

To evaluate the pathological changes in the aortic root of atherosclerotic mice, sections of aortic tissue were stained with H&E and Oil Red O. As shown in Figure 1 A1/B1, the normal aortic structure exhibited normal architecture without pathological changes. The intimal layer remained intact without hyperplasia, and the lumen showed no signs of stenosis. In the tunica media, smooth muscle cells and elastic fibers were orderly arranged, with preserved cellular morphology and extracellular matrix composition. In the model group (Figure 1 A2/B2), the aortic intima exhibited pronounced intimal thickening, accompanied by the formation of a complex atherosclerotic plaque. There were abundant lipid-laden foam cells accumulated within the subintimal layer, interspersed with cholesterol crystals and amorphous lipid deposits. A central necrotic core, comprising cellular debris and necrotic material, was encircled by collagen fiber bundles and a band of inflammatory infiltrates. The lesion was overlaid by a dense fibroconnective tissue layer, forming a well-defined fibrous cap. In 2.5 mg/kg AVT group (Figure 1 A3/B3) and 12, 24, 48 mg/kg GC groups (Figure 1 A4-6/B4-6), the aortic intima showed significantly reduced pathological thickening, accompanied by decreased plaque area (Figure 1 A7/B7). Inflammatory cell recruitment to the lesion site was markedly attenuated, and luminal narrowing was significantly alleviated, suggesting improved vascular patency.

Dyslipidemia constitutes a major modifiable risk factor for atherosclerotic cardiovascular disease. GC’s lipid-modulating effects were assessed via TG/TC/LDL-C/HDL-C quantification across experimental groups (Figure 1C). Compared to the control group, the model group exhibited significantly elevated serum levels of TG (5.14 ± 0.52 mM), TC (40.63 ± 2.17 mM), and LDL-C (16.58 ± 0.82 mM) (all *P* < 0.01), whereas HDL-C was markedly reduced (2.20 ± 0.67 mM, *P* < 0.01). Both AVT and GC significantly improved the blood lipid levels (all *P* < 0.01 vs. model group). The results showed that GC demonstrated lipid-lowering properties and suppressed atherosclerotic progression.

### GC enhances autophagy in AS model mice

**FIGURE 2:**
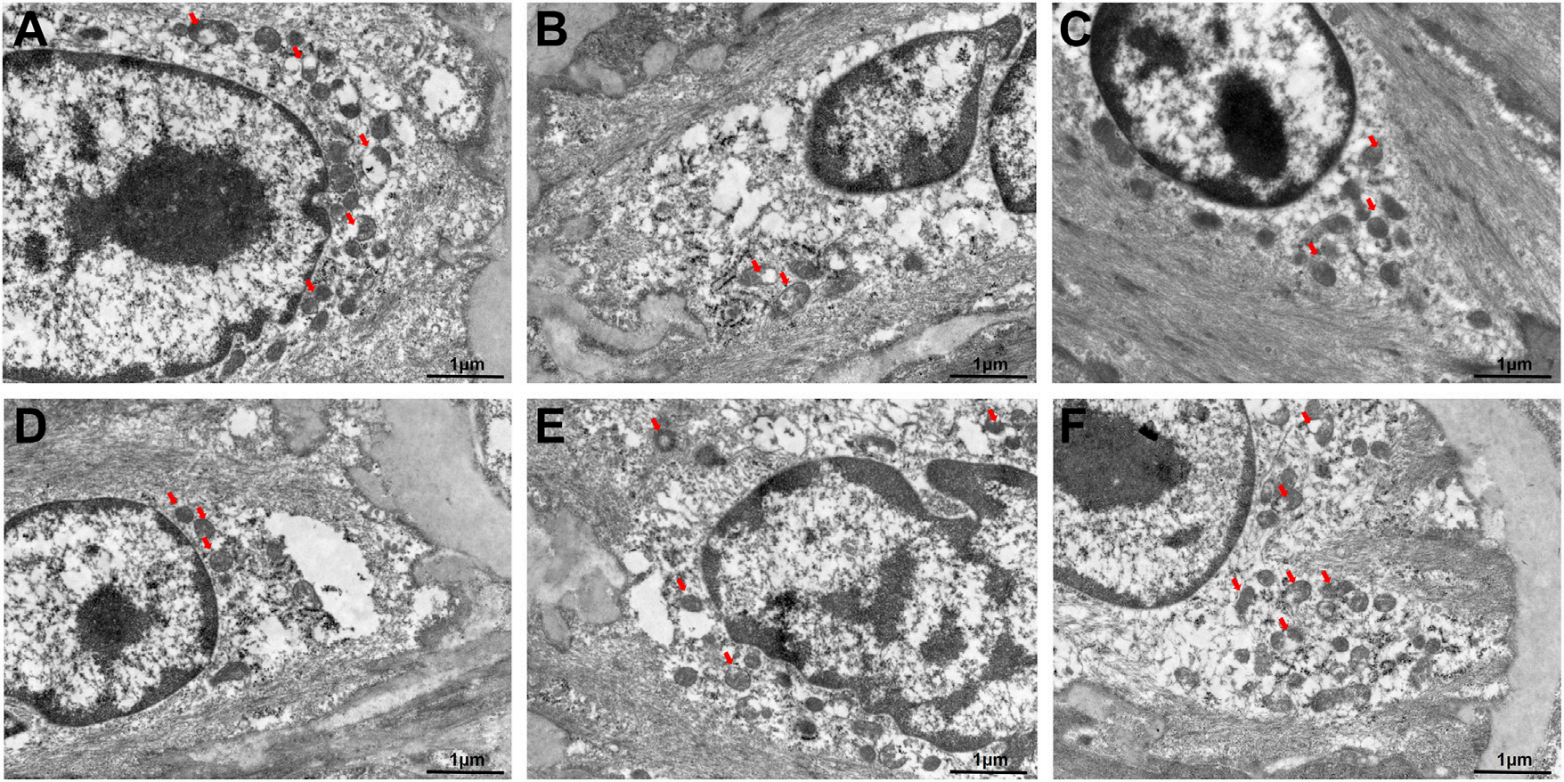
Transmission electron microscope for the autophagosomes in AS model mice. **(A)** Control group, **(B)** model group, **(C)** 2.5 mg/kg AVT group, **(D)** 12 mg/kg GC group, **(E)** 24 mg/kg GC group, **(F)** 48 mg/kg GC group.

In addition, the autophagosome formation was ultrastructurally characterized using TEM. As shown in Figure 2A, the autophagosome was significantly discovered in control group. In the model group, TEM revealed impaired autophagy in atherosclerotic aortas, characterized by accumulated autophagosomes (double-membrane vesicles) within macrophages and smooth muscle cells, often containing undegraded lipid droplets and cellular debris (Figure 2B). Our data confirmed that 2.5 mg/kg AVT slightly increased the expression of autophagosome in AS model mice (Figure 2C). Following treatment by 12, 24, 48 mg/kg GC, the amount of autophagosome was significantly increased (Figures 2D–F). These results suggested that GC could diminish lipid-engorged autophagic vacuoles and attenuate autophagosome-lysosome fusion impairment. The study indicated that GC might alleviate AS by promoting autophagy.

### GC inhibits inflammation and regulates inflammatory cytokines expression in AS model mice

**FIGURE 3:**
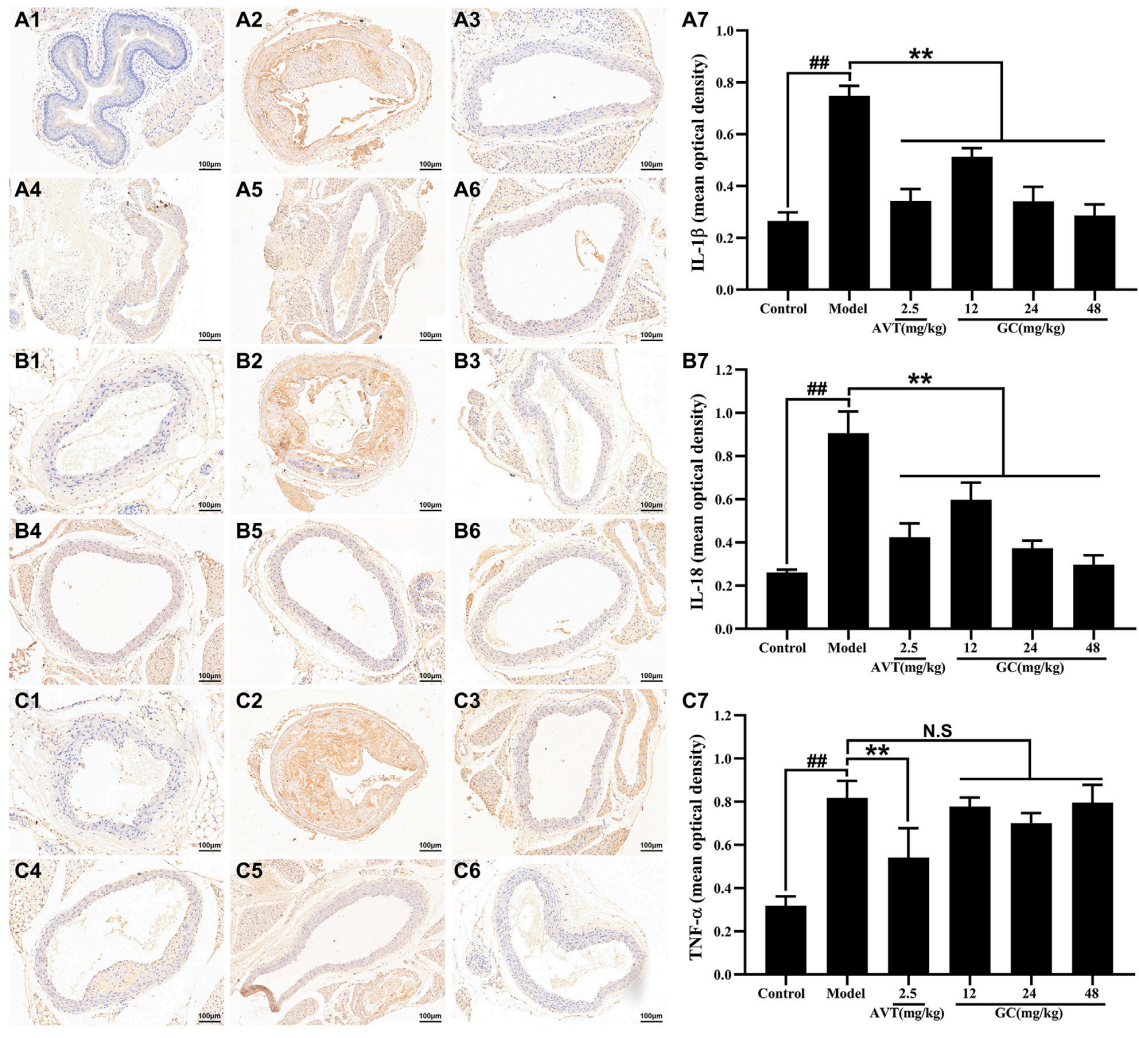
Effect of GC on inflammatory cytokines expression in AS model mice. Immunohistochemistry was applied to determine the expression of IL-1β **(A)**, IL-18 **(B)** and TNF-α **(C)**. (A1/B1/C1) Control group, (A2/B2/C2) model group, (A3/B3/C3) 2.5 mg/kg AVT group, (A4/B4/C4) 12 mg/kg GC group, (A5/B5/C5) 24 mg/kg GC group, (A6/B6/C6) 48 mg/kg GC group. (A7/B7/C7) Mean optical density of IL-1β, IL-18 and TNF-α. Data were expressed as mean ± SD (n = 6). ^##^ *P* < 0.01 vs. control group; ^∗^ *P* < 0.05, ^∗∗^ *P* < 0.01 vs. model group.

The progression of atherosclerotic lesions is closely linked to inflammatory mechanisms, forming a self-amplifying cycle between both processes. Inflammatory cytokines, such as TNF-α, IL-6, and IL-18, play pivotal roles in promoting the development of AS. Among these. Among these, IL-1β emerges as a particularly destructive mediator, driving vascular inflammation through mechanisms such as endothelial activation, leukocyte recruitment, and plaque destabilization. The central role of IL-1β in inflammasome signaling cascades further establishes it as a critical therapeutic target for interrupting the inflammatory feedback loop in AS. We therefore used immunohistochemistry to examine the expression of key inflammatory cytokines in aortic root tissues (Figure 3). Baseline expression levels of IL-1β, IL-18, and TNF-α were low in the control group. After modeling, the expression levels of IL-1β, IL-18, and TNF-α were significantly increased by 2.82-, 3.46-, and 2.57-fold, respectively, compared with the control group (all *P* < 0.01). Treatment with 2.5 mg/kg AVT significantly reduced the expression of IL-1β, IL-18, and TNF-α (all *P* < 0.01 vs. model group). Furthermore, administration of 12, 24, and 48 mg/kg GC reduced the expression of IL-1β (0.51 ± 0.04, 0.34 + 0.06, 0.29 ± 0.05, mean optical density) and IL-18 (0.60 ± 0.09, 0.37 + 0.04, 0.30 ± 0.05, mean optical density), but did not significantly affect TNF-α levels (0.78 ± 0.05, 0.70 + 0.05 0.80 ± 0.09, mean optical density).

### GC elevates LAMP-2A expression and inhibits NLRP3 inflammasome expression in AS model mice

**FIGURE 4:**
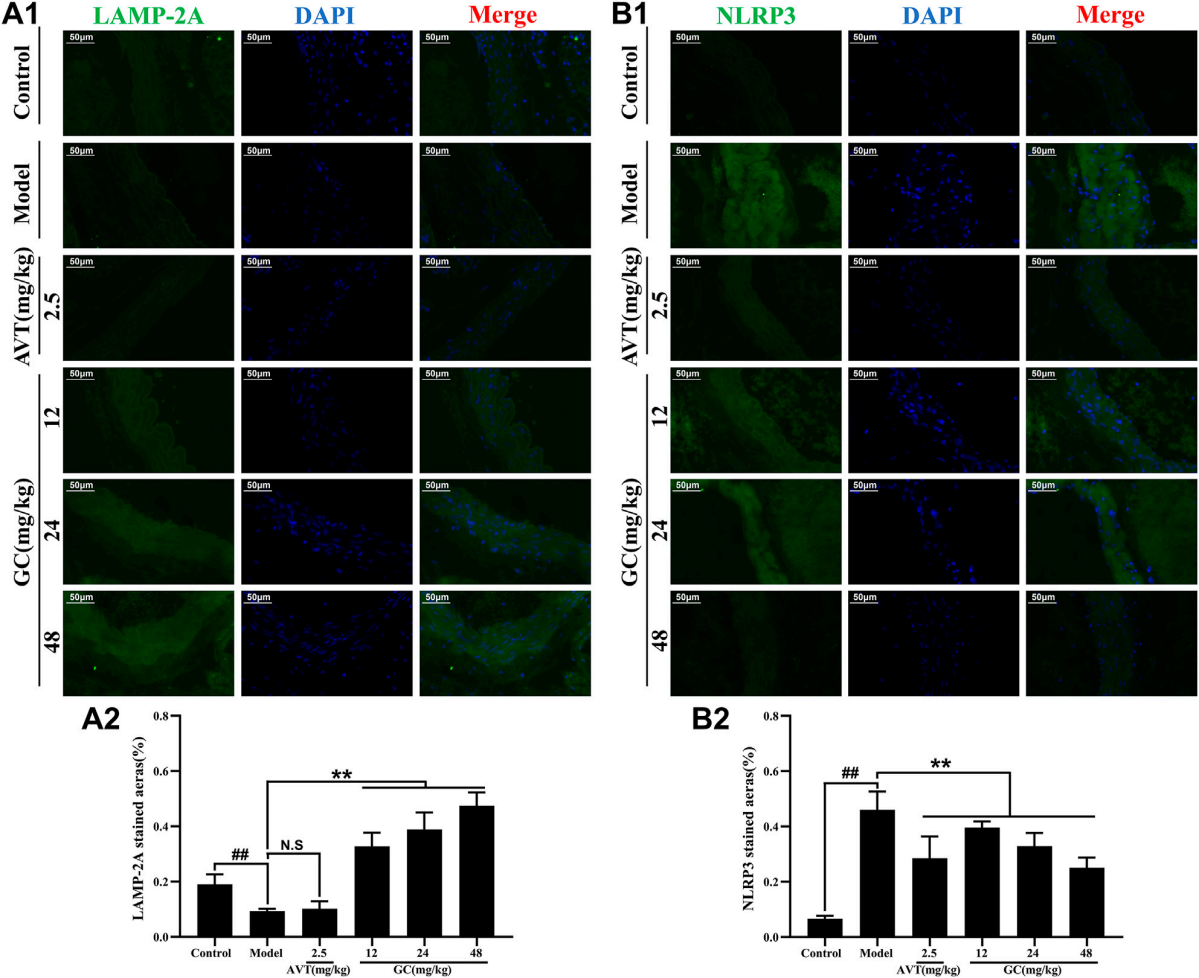
Effect of GC on LAMP-2A and NLRP3 inflammasome expressions in AS model mice. Immunofluorescence staining was performed for measurements of LAMP-2A (A1) and NLRP3 (B1). Quantitative analysis of LAMP-2A (A2) and NLRP3 (B2). Data were expressed as mean ± SD (n = 6). ^##^ *P* < 0.01 vs. control group; ^∗^ *P* < 0.05, ^∗∗^ *P* < 0.01 vs. model group.

LAMP-2A serves as the rate-limiting protein in CMA functionality. Our prior research revealed that GC acts as a selective LAMP-2A agonist, enhancing LAMP-2A expression in a dose-dependent manner (Data not shown). The NLRP3 inflammasome/IL-1β-dependent inflammatory response plays a pivotal role in the pathogenesis and progression of AS. We therefore examined the protein expression of LAMP-2A and the NLRP3 inflammasome in aortic tissues using immunofluorescence staining. As shown in Figure 4A, LAMP-2A expression in the control group was slightly low. However, there was no significant difference between control and model group. Moreover, LAMP-2A expression in 2.5 mg/kg AVT group was not significantly increased compared with the control group (*P* > 0.5). But 12, 24, and 48 mg/kg GC all obviously upregulated LAMP-2A expression level (3.5, 4.2 and 5.1-fold, all *P* < 0.01 vs. model group). It was shown in Figure 4B that NLRP3 inflammasome expression in the control group was low but was obviously increased after modeling (*P* < 0.01 vs. control group). AVT and 12, 24, 48 mg/kg GC could significantly inhibit NLRP3 inflammasome expression. These findings indicate that GC enhances LAMP-2A expression, thereby potentiating CMA activity and promoting the degradation of the NLRP3 inflammasome.

### GC protects against inflammatory insult in LPS/ATP stimulated RAW264.7 cells

**FIGURE 5:**
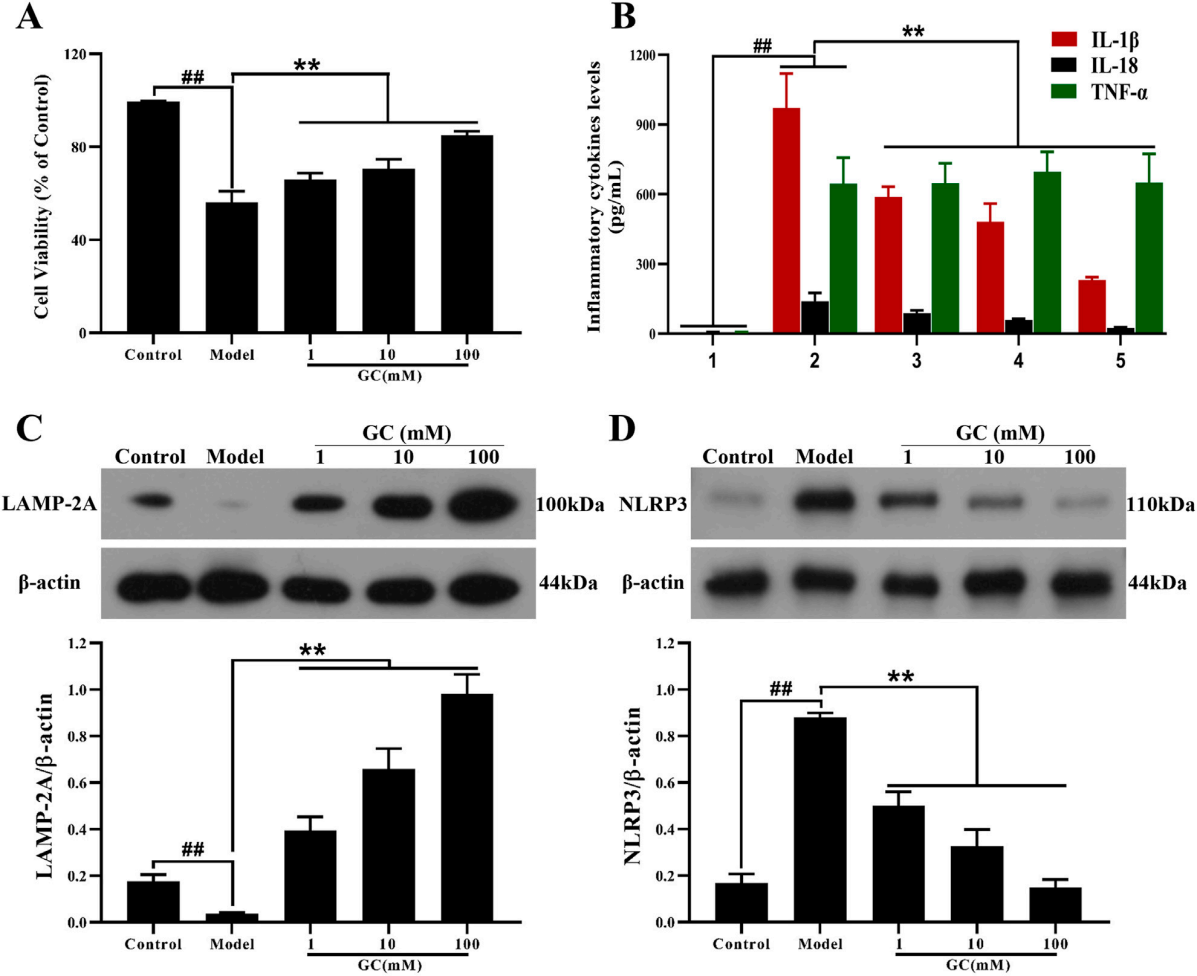
Effect of GC on cell viability, supernatant inflammatory cytokines levels and expressions of LAMP-2A, NLRP3 inflammasome in LPS/ATP stimulated RAW264.7 cells. MTT assay was performed for measurement of cell viability **(A)**. ELISA kits were used for measurement of IL-1β, IL-18 and TNF-α **(B)**. Western blot was employed to quantify the expressions of LAMP-2A **(C)**, NLRP3 **(D)**. Results were expressed as Protein/reference protein ratio. Data were expressed as mean ± SD (n = 3). ^##^ *P* < 0.01 vs. control group; ^∗^ *P* < 0.05, ^∗∗^ *P* < 0.01 vs. model group.

As shown in Figure 5A, LPS/ATP injured RAW264.7 cells elicited significant attenuation of cell viability (56.24% ± 4.71%, *P* < 0.01 vs. control group) as assessed by MTT assay. GC preconditioning (1, 10, 100 mM) demonstrated significant enhancement of RAW264.7 cell viabilities (66.10% ± 2.66%, 70.54% ± 4.07%, 85.10% ± 1.66%, *P* < 0.01 vs. model group).

### GC inhibits inflammatory cytokines expression in LPS/ATP stimulated RAW264.7 cells

The progression of AS is characterized by progressive pathological changes, driven largely by the expression of inflammatory cytokines that initiate a self-amplifying inflammatory cascade. To further investigate the inflammatory response, we examined the expression of inflammatory cytokines (IL-1β, IL-18, TNF-α) in RAW264.7 macrophages following inflammatory injury induced *in vitro* by LPS/ATP stimulation (Figure 5B). LPS/ATP stimulation triggered the activation of inflammatory responses (IL-1β, IL18 and TNF-α; all *P* < 0.01 vs. control group), whereas treatment of 1, 10, and 100 μM GC validly reduced the amount of IL-1β, IL18 (all *P* < 0.01 vs. model group) without TNF-α.

### GC enhances expression of LAMP-2A and inhibits overexpression of NLRP3 in LPS/ATP stimulated RAW264.7 cells

LAMP-2A, a key receptor and regulator of CMA, regulates macrophage function. LAMP-2A–mediated CMA facilitates the degradation of NLRP3 inflammasome components, thereby suppressing the release of IL-1β and IL-18 and leading to attenuation of the inflammatory cascade. Furthermore, we investigated the impact of GC on LAMP-2A and NLRP3 expression levels in RAW264.7 cells stimulated with LPS/ATP. As shown in Figure 5C, the expression level of LAMP-2A were relatively low in the control group. After LPS/ATP stimulation, LAMP-2A expression did not increase, suggesting a deficiency in CMA activity that may contribute to disease progression. Notably, treatment with GC at concentrations of 1, 10 and 100 μM remarkably enhanced both the activation and expression of LAMP-2A (9.9, 16.5 and 24.6-fold, all *P* < 0.01 vs. model group), suggesting its potential to restore CMA efficiency and counteract lysosomal dysfunction in inflammatory contexts. Consistent with *in vivo* findings, treatment with GC at concentrations of 1, 10, and 100 μM significantly suppressed NLRP3 inflammasome expression in LPS/ATP-induced inflammatory injury, highlighting its capacity to disrupt inflammasome assembly and attenuate downstream pro-inflammatory signaling (Figure 5D).

#### GC exhibits no regulatory influence on NF-κB and MAPK signaling pathways

**FIGURE 6:**
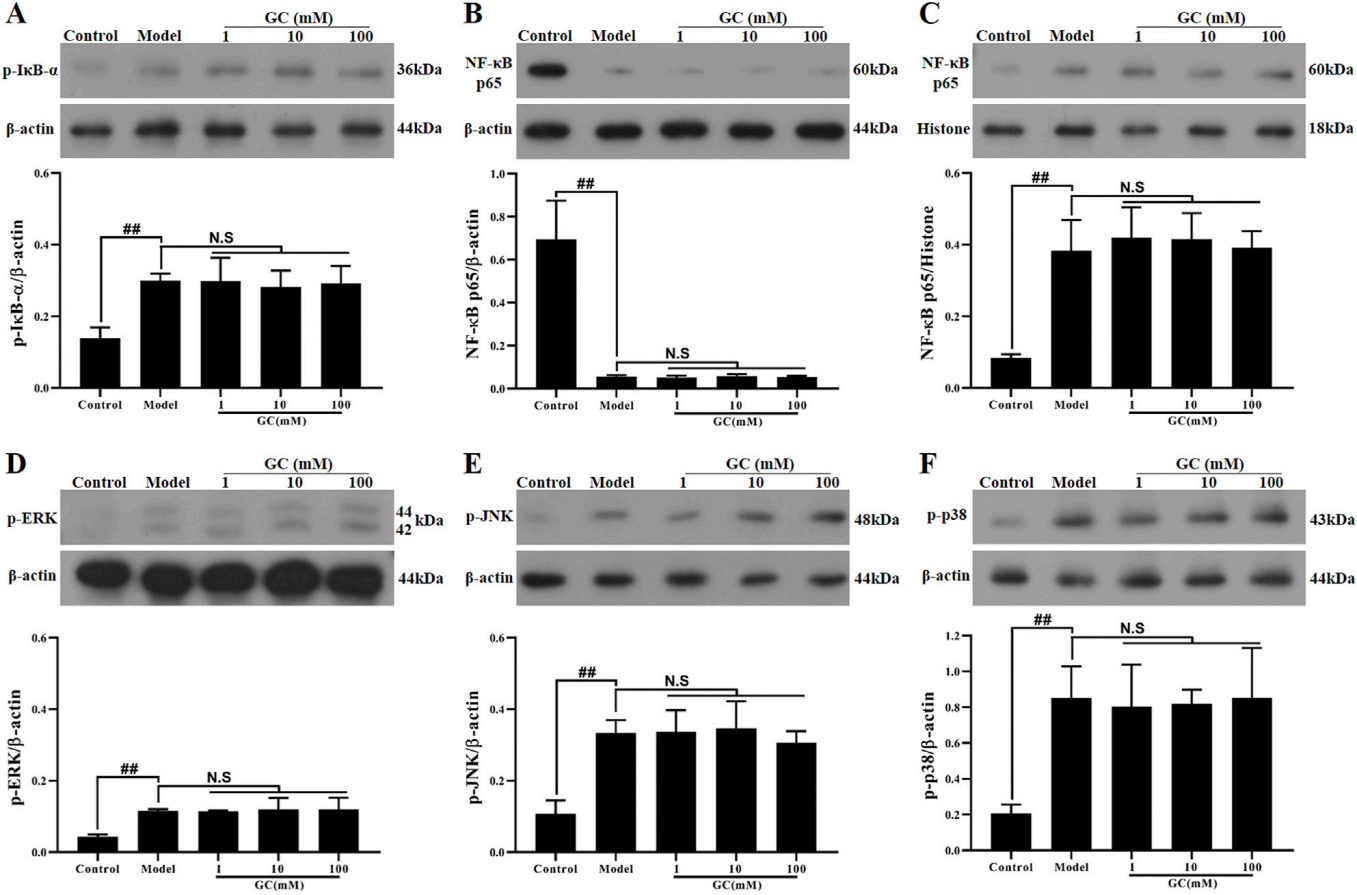
Effect of GC on expressions of p-IκB-α, NF-κB p65, p-ERK, p-JNK and p-p38 in LPS/ATP stimulated RAW264.7 cells. Western blot was employed to quantify the expressions of p-IκB-α **(A)**, cytoplasmic NF-κB p65 **(B)**, nuclear NF-κB p65 **(C)**, p-ERK **(D)**, p-JNK **(E)** and p-p38 **(F)**. Results were expressed as Protein/reference protein ratio. Data were expressed as mean ± SD (n = 3). ^##^ *P* < 0.01 vs. control group; ^∗^ *P* < 0.05, ^∗∗^ *P* < 0.01 vs. model group.

The NLRP3 inflammasome forms a tightly interconnected regulatory network with the NF-κB and MAPK signaling pathways, collectively orchestrating the inflammatory response. We therefore investigated the regulatory effects of GC on NLRP3 inflammasome expression within the NF-κB/MAPK signaling axis. The data presented in Figure 6 demonstrated that LPS/ATP stimulation significantly elevated the phosphorylation of IκB-α, JNK, ERK, p38 MAPK and activated the NF-κB pathway (91.4% decrease in cytoplasmic p65 levels and 4.8-fold increase in nuclear p65 levels compared to control group) relative to the control group, indicating coordinated activation of the NF-κB and MAPK signaling pathway. However, following the addition of 1, 10 and 100 μM GC, the activation trends of both the NF-κB and MAPK signaling pathways remained unaltered, suggesting that GC’s anti-inflammatory effects may be mediated through mechanisms independent of these canonical pathways, such as direct modulation of NLRP3 inflammasome assembly or enhancement of CMA activity.

### LAMP-2A gene is successfully and stably silenced in RAW264.7 cells

**FIGURE 7:**
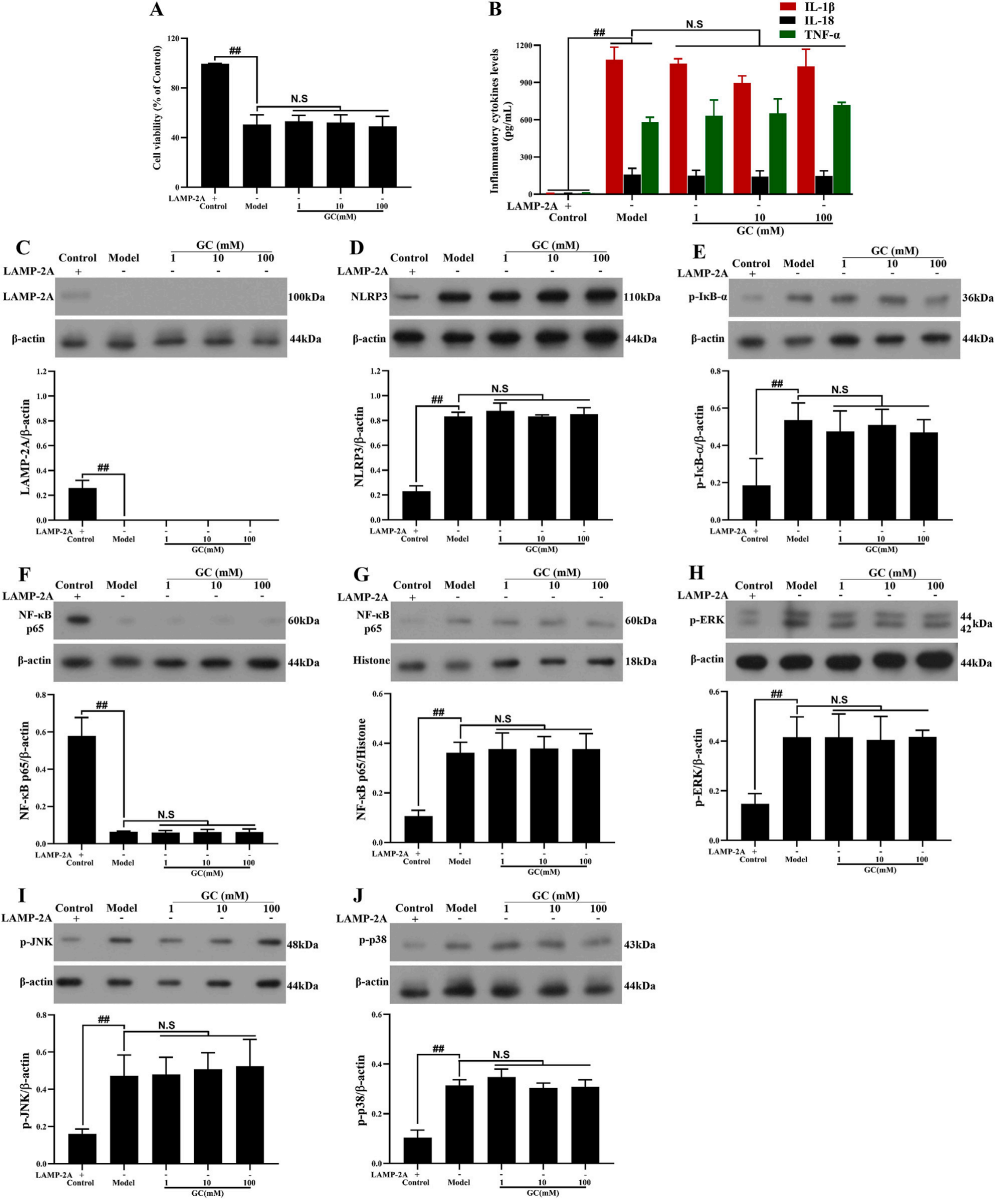
Effect of GC on cell viability, supernatant inflammatory cytokines and expressions of LAMP-2A, NLRP3 inflammasome, NF-κB/MAPK signaling pathway proteins in LPS/ATP stimulated RAW264.7 cells after LAMP-2A gene silencing. MTT assay was performed for measurement of cell viability **(A)**. ELISA kits were used for measurement of IL-1β, IL-18 and TNF-α **(B)**. Western blot was employed to quantify the expressions of LAMP-2A **(C)**, NLRP3 inflammasome **(D)**, p-IκB-α **(E)**, cytoplasmic NF-κB p65 **(F)**, nuclear NF-κB p65 **(G)**, p-ERK **(H)**, p-JNK **(I)** and p-p38 **(J)**. Results were expressed as Protein/reference protein ratio. Data were expressed as mean ± SD (n = 3). ^##^ *P* < 0.01 vs. control group; ^∗^ *P* < 0.05, ^∗∗^ *P* < 0.01 vs. model group.

In the control group, RAW264.7 cells transfected with the pGPU6/Hygro plasmid exhibited negligible LAMP-2A expression, whereas pretreatment with the pGPU6/Hygro-LAMP-2A construct achieved sustained and effective suppression of LAMP-2A levels across experimental groups (Figure 7B).

GC cannot enhance cell viability, inhibit the expressions of NLRP3 inflammasome and inflammatory cytokines and activate NF-κB/MAPK signaling pathways after LAMP-2A gene silence.

To further validate whether GC exerts its atheroprotective effects by positively regulating CMA in macrophages-thereby reducing NLRP3 inflammasome expression and mitigating inflammatory injury-we employed LAMP-2A gene silencing to disrupt CMA functionality. The results in Figure 7 demonstrated that 1, 10 and 100 μM GC all exhibited no capacity to promote cell viability, suppress NLRP3 inflammasome and inflammatory cytokines (IL-1β, IL18 and TNF-α) release and activate NF-κB/MAPK signaling pathways against LPS/ATP injury after LAMP-2A gene was silenced.

## Discussion

AS is a progressive vascular disorder driven by two core pathological mechanisms: systemic lipid metabolism dysregulation and sustained inflammatory responses (Groenen et al., 2021). During AS progression, disordered lipid metabolism propels a state of chronic inflammation. Concurrently, inflammatory signals perpetuate this process by impairing lipid clearance and intensifying oxidative stress, culminating in a synergistic, self-amplifying cycle that advances plaque growth and vascular injury (Ajoolabady et al., 2024). This bidirectional interaction establishes a self-reinforcing loop wherein inflammatory mediators both drive and are amplified by the advancement of arterial lesions.

*Ginkgo biloba*, a relict gymnosperm and the sole extant species of the family Ginkgoaceae, has an evolutionary history dating back over 270 million years, with no closely related extant species. Its leaf extracts contain a variety of pharmacologically active constituents, such as flavonoid glycosides (approximately 24%) and terpenoid lactones (∼6%, including ginkgolides A, B, C, and J, as well as bilobalide), which exhibit multi-target therapeutic properties (Eisvand et al., 2020; Barbalho et al., 2022). Among these bioactive constituents, we have prioritized preclinical investigations on ginkgolide B (GB) and GC. Preliminary findings demonstrate their significant therapeutic efficacy against both myocardial and cerebral ischemia-reperfusion injuries, with mechanistic studies revealing these effects are primarily mediated through their potent anti-inflammatory properties (Zhang et al., 2018; Li et al., 2023). The results of this study demonstrate that GC not only significantly ameliorates pathological phenotypes in an AS model-such as reducing aortic plaque formation, lipid deposition, and inflammatory infiltration-but also comprehensively improves lipid metabolism, indicating its potential for multi-target intervention in AS. Notably, the ability of GC to enhance vascular integrity and inhibit foam cell formation suggests that it may target two central processes in AS pathogenesis: endothelial dysfunction and macrophage-derived foam cell formation. Of course, this study has certain limitations. For instance, the specific molecular targets of GC and the precise mechanisms by which it regulates the crosstalk between lipid metabolism and inflammatory pathways have not been fully elucidated. Additionally, since these findings are primarily derived from animal and cellular models, the efficacy of their clinical translation requires further validation.

Macrophages constitute the predominant cellular population within atherosclerotic plaques and constitute a major contributor to AS etiology (Hou et al., 2023). The transformation of macrophages into foam cells via pathological lipid uptake is a pivotal event in atherosclerotic lesion formation, driving plaque progression by increasing lipid accumulation, sustaining inflammation, and destabilizing plaques through MMP-mediated degradation of the extracellular matrix. Emerging AS therapies prioritize targeting foam cell lipid depletion-dual-targeting cholesterol metabolism and inflammation synergistically reduces plaque vulnerability. CMA, a lysosomal degradation pathway regulating cellular glucose and lipid homeostasis, has been implicated in AS pathogenesis through its functional impairment (Yao and Shen, 2023). However, the therapeutic potential of GC to modulate CMA activity, particularly its effects on LAMP-2A translocation efficiency, remains uncharacterized. Using TEM, we found that atherosclerotic mice exhibited a failure in autophagosome-lysosome fusion and an accumulation of undegraded autophagosomes in vascular cells, indicating autophagic flux impairment. However, GC effectively reversed these ultrastructural pathologies. While autophagy dysfunction is implicated in AS, few therapies directly target autophagosome-lysosome fusion. GC’s ability to achieve this-documented here via direct TEM evidence-represents a novel mechanistic insight and distinguishes it from conventional lipid-lowering or anti-inflammatory strategies. It suggests that GC operates as a multi-scale regulator: improving systemic lipid metabolism while also resolving core cellular pathologies. In addition, we established an *in vitro* RAW264.7 macrophage model co-stimulated with LPS and ATP to recapitulate the inflammatory injury phenotype of macrophages in atherosclerotic disease. While LPS/ATP exposure reduced RAW264.7 macrophage viability, GC preconditioning enhanced cell survival. Thus, while GC exerts protective effects against AS-induced macrophage injury, its precise molecular mechanisms require further elucidation.

LAMP-2A is the key and specific receptor protein in CMA, responsible for mediating the transport of selective substrate proteins across the lysosomal membrane into the lysosomal lumen for degradation. Following the formation of atherosclerotic lesions, impaired CMA in macrophages-frequently caused by LAMP-2A deficiency-promotes the accelerated progression of plaques (Valdor and Martinez-Vicente, 2024). Our data indicated that GC significantly upregulates LAMP-2A expression in the aortic tissues of atherosclerotic mice. *In vitro* findings similarly revealed that GC could significantly enhance the expression of LAMP-2A, thereby improving the autophagic function of macrophages. Our study identifies that GC attenuates AS by boosting LAMP-2A expression and CMA in vascular cells. This enhanced protein quality control provides a unified mechanism for GC’s ability to alleviate inflammation and lipid accumulation, highlighting CMA as a potential therapeutic target. Although comprehensive CMA evaluation typically requires substrate flux assays, our observed LAMP-2A induction magnitude aligns with established thresholds for predicting CMA activation in nutrient-stress contexts (Park et al., 2022). Subsequent studies will employ CMA-specific substrate assays to directly quantify CMA kinetics.

Emerging evidence indicates that AS progression is critically driven by NLRP3 inflammasome/IL-1β signaling, highlighting its role as a chronic inflammatory disease. CANTOS trial established IL-1β as a pivotal therapeutic target for anti-inflammatory strategies in AS (Ridker et al., 2017). The NLRP3 inflammasome functions as a key upstream regulator of IL-1β production and acts as a central organizer of inflammatory and immune processes. Its activation and subsequent IL-1β release are observed beginning in early-stage atherosclerosis and continue to sustain a pro-inflammatory environment throughout disease development (Olsen et al., 2022). Intriguingly, CMA, a highly efficient and substrate-specific protein degradation system, modulates NLRP3 inflammasome activation and downstream IL-1β expression via its proteolytic regulatory network (Abbate et al., 2020). We accordingly investigated how GC regulates the NLRP3 inflammasome and key inflammatory cytokines (IL-1β, IL-18, TNF-α) in the aortic tissue of atherosclerotic mice. Treatment with GC exhibited a clear effect against AS-induced inflammation via obvious suppression of the NLRP3 inflammasome and downstream inflammatory cytokines IL-1β, IL18. However, GC exerts no significant effect on TNF-α expression, indicating that it directly targets the NLRP3 inflammasome rather than its upstream proteins. Moreover, *in vitro* experimental data further confirmed that GC effectively suppressed NLRP3 inflammasome assembly and attenuated pro-inflammatory cytokine release. The selective suppression of the NLRP3 inflammasome and its downstream cytokines (IL-1β and IL-18), but not TNF-α, by GC provides critical insight into its mechanism of anti-inflammatory action in AS. TNF-α is primarily regulated by NF-κB signaling, which operates upstream of NLRP3 activation as a priming signal. The fact that GC did not alter TNF-α levels suggests that its inhibitory effect is not mediated through broad suppression of upstream inflammatory priming, but rather through specific disruption of NLRP3 inflammasome assembly and activation. This specificity is significant therapeutically, as global immunosuppression often leads to adverse effects, whereas targeted NLRP3 inhibition may offer a more favorable safety profile. This finding positions GC as a selective NLRP3 pathway inhibitor rather than a general anti-inflammatory agent. The concordance between *in vivo* and *in vitro* data strengthens the conclusion that GC directly interferes with the NLRP3 complex formation, potentially through modulating key components. Given the central role of NLRP3-driven inflammation in atherosclerotic plaque progression and vulnerability, GC’s specific targeting of this pathway provides a mechanistic basis for its observed atheroprotective effects. However, our current dataset cannot definitively distinguish between CMA and ubiquitin-proteasome mediated degradation of NLRP3. Future studies will prioritize pharmacological perturbations with MG132 to resolve this mechanistic ambiguity, particularly under varying metabolic stress conditions. Furthermore, this action may synergize with GC’s previously reported benefits on autophagy and lipid metabolism, collectively contributing to vascular protection. Future studies should focus on identifying the direct molecular target of GC within the NLRP3 inflammasome complex to fully elucidate its mechanism of action.

Despite the recognized role of the NLRP3 inflammasome in AS, several key controversies present challenges for therapeutic development. Firstly, the primacy of specific activation pathways (e.g., ion flux vs. lysosomal rupture) in vascular cells remains debated. Secondly, and more critically, a major hurdle lies in achieving sufficient specificity to inhibit NLRP3-driven pathology without incurring broad immunosuppression, a common limitation of existing anti-inflammatory therapies. This has spurred the search for novel mechanisms to modulate NLRP3 activity with greater precision.

The activation of the NLRP3 inflammasome is mechanistically linked to upstream regulatory signaling pathways, including NF-κB and MAPK (Li et al., 2020; Yuan et al., 2022). To validate that GC attenuates the release of pro-inflammatory cytokines (e.g., IL-1β, IL-18) through restoring LAMP-2A-dependent CMA and suppressing NLRP3 inflammasome assembly, we further investigated the modulatory effects of GC on NF-κB and MAPK signaling cascades. Our results demonstrated GC reduces NLRP3 inflammasome activation through a unique mechanism independent of NF-κB or MAPK signaling. Instead, GC enhances autophagic clearance, ameliorates lysosomal impairment, and removes endogenous triggers of inflammation-such as cellular debris and damaged organelles-that drive NLRP3 activation. This autophagy-focused action not only suppresses harmful inflammation but also targets the underlying cellular damage, highlighting GC’s potential as a targeted therapy for chronic inflammatory diseases like AS.

To conclusively validate whether the atheroprotective effects of GC are mediated through LAMP-2A-specific transcriptional activation, a mechanism involving CMA functional restoration, suppression of NLRP3 inflammasome hyperactivation, and attenuation of NLRP3/IL-1β-driven inflammatory cascades, we performed LAMP-2A genetic knockdown in macrophages using siRNA-mediated silencing under standardized LPS/ATP co-stimulation conditions. Following LAMP-2A silencing, GC is unable to rescue macrophage viability, suppress NLRP3 inflammasome activation, or attenuate IL-1β/IL-18 release under LPS/ATP stimulation. In addition, GC’s lack of effect on NF-κB/MAPK signaling persisted even after LAMP-2A knockdown. However, future studies employing LAMP-2A overexpression will be valuable to further solidify this mechanism. LAMP-2A knockdown completely abolished GC’s ability to rescue macrophage viability and suppress NLRP3 inflammasome activation, establishing LAMP-2A as an essential mediator of GC’s protective effects. These results confirm that GC alleviates inflammation specifically through enhancing LAMP-2A-dependent CMA, rather than through NF-κB/MAPK signaling or non-specific mechanisms. By restoring CMA, GC promotes clearance of inflammatory triggers leading to NLRP3 inhibition, revealing a precise mechanism for its anti-atherosclerotic action. Future studies with LAMP-2A overexpression would further solidify this pathway as a therapeutic target.

The central and novel finding of this study is the identification of the LAMP-2A/CMA pathway as the key mechanism through which GC mitigates atherosclerotic injury by promoting NLRP3 inflammasome degradation. While natural compounds like GC often exhibit pleiotropic effects, our data within the scope of this model indicate that modulation of the NF-κB pathway is not a principal contributor to the observed protection. Nonetheless, contributions from other pathways, such as antioxidant activity which has been reported for GC in other disease models (Zhang et al., 2022), could potentially serve as complementary mechanisms. Further investigations are warranted to explore these potential ancillary effects and their possible synergy with the CMA mechanism we have elucidated.

Our study is the first to demonstrate that GC mitigates NLRP3 activation through a novel LAMP-2A-mediated CMA pathway. This discovery establishes a previously unrecognized mechanistic link between proteostasis and inflammasome regulation in AS. Unlike broad-spectrum inhibitors, our proposed strategy-therapeutically enhancing a specific proteostatic pathway-presents a novel approach that effectively circumvents the critical issue of immunosuppression. While the precise degradation routes require further validation, these findings suggest Ubiquitin-Proteasome system-mediated clearance as a plausible regulatory checkpoint, meriting targeted interrogation in subsequent work.

Research on natural products and monomers from traditional medicine underscores the potential of targeting NLRP3 in AS (Wei et al., 2023; Zhu et al., 2024a). Our findings demonstrate that the natural monomer GC inhibits NLRP3 via the LAMP-2A/CMA pathway, providing a novel and specific mechanism compared to the broader anti-inflammatory effects of many natural compounds. This raises the possibility that other natural compounds may also act through CMA, suggesting a new direction for future research. Moreover, this study elucidates a previously unrecognized mechanism underlying the bioactivity of GC, while simultaneously advancing the conceptual framework through which natural compounds elicit targeted therapeutic outcomes via specific molecular pathways (Zhu et al., 2024b; Zhu et al., 2025). Thus, our work establishes GC as a mechanistically elucidated candidate and offers a new framework for understanding natural product efficacy.

**FIGURE 8:**
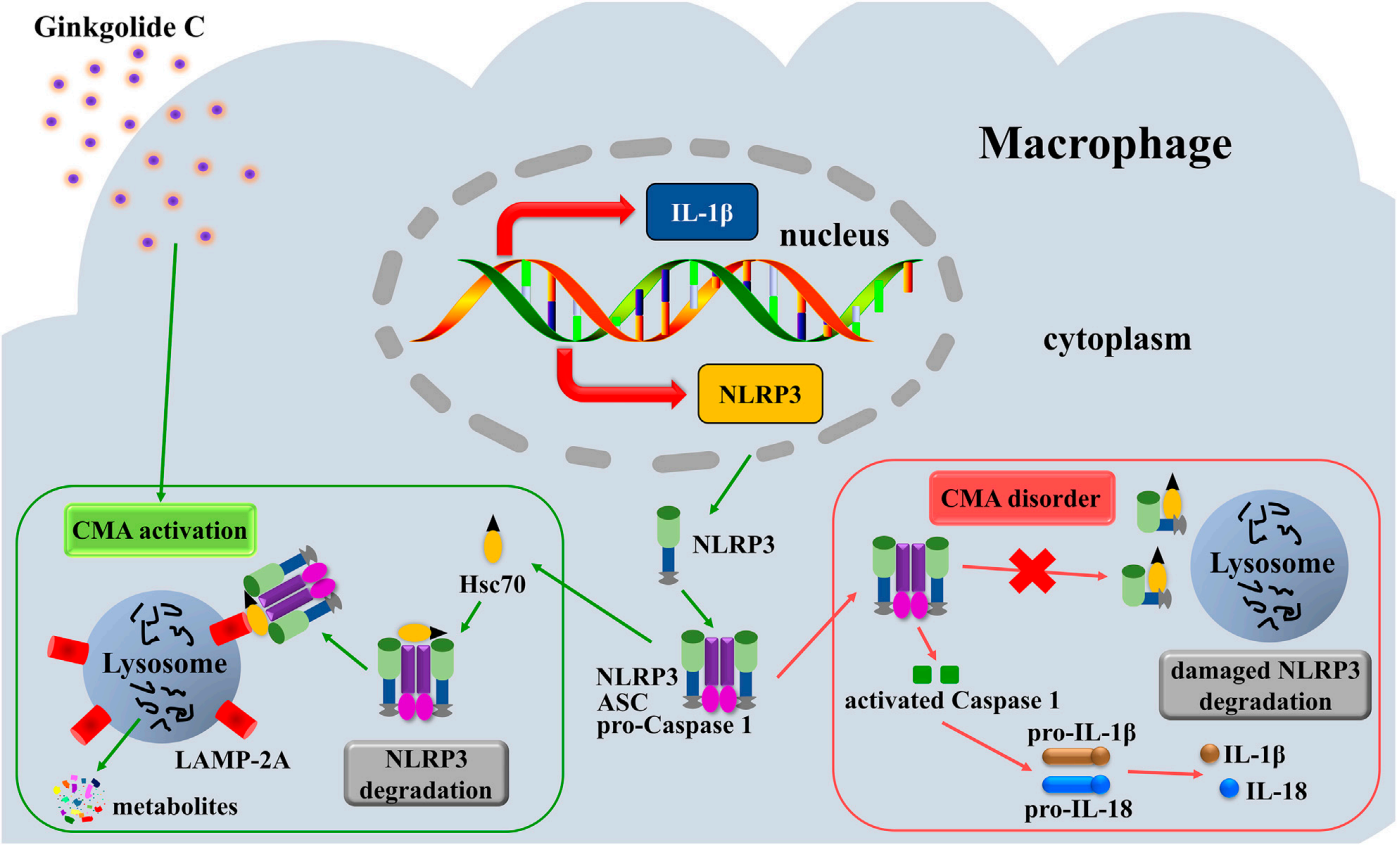
Mechanistic illustration of GC-mediated suppression of AS pathogenesis.

In conclusion, this study demonstrates that GC augments CMA in macrophages, mechanistically linking this enhancement to selective NLRP3 inflammasome degradation via lysosomal proteolytic clearance, which consequently suppresses NLRP3/IL-1β inflammatory signaling and ameliorates vascular endothelial dysfunction (Figure 8).

## Data Availability

The original contributions presented in the study are included in the article/supplementary material, further inquiries can be directed to the corresponding author.
